# Improved patient-reported outcomes in patients with psoriatic arthritis treated with abatacept: results from a phase 3 trial

**DOI:** 10.1186/s13075-018-1769-7

**Published:** 2018-12-06

**Authors:** Vibeke Strand, Evo Alemao, Thomas Lehman, Alyssa Johnsen, Subhashis Banerjee, Harris A. Ahmad, Philip J. Mease

**Affiliations:** 1 Division of Immunology/Rheumatology, Stanford University School of Medicine, Palo Alto, CA USA; 2 Bristol-Myers Squibb, Princeton, NJ USA; 3 Swedish Medical Center and University of Washington, Seattle, WA USA

**Keywords:** DMARDs (biologic), Outcomes research, Patient perspective, Psoriatic arthritis

## Abstract

**Background:**

To explore the effect of abatacept treatment on patient-reported outcomes (PROs) in psoriatic arthritis (PsA).

**Methods:**

Patients with PsA were randomised (1:1) to subcutaneous abatacept 125 mg weekly/placebo for 24 weeks with early escape (EE) to open-label abatacept (week 16). Adjusted mean changes from baseline to weeks 16 (all patients) and 24 (non-EE responders) in Health Assessment Questionnaire-Disability Index (HAQ-DI), Short Form-36 (SF-36; physical and mental component summary and domains), Dermatology Life Quality Index and Functional Assessment of Chronic Illness Therapy-Fatigue (FACIT-F) were evaluated. Subpopulations were analysed by baseline C-reactive protein (CRP) level (> vs ≤ upper limit of normal [ULN]) and prior tumour necrosis factor inhibitor (TNFi) exposure. Proportions of patients reporting improvements ≥ minimal clinically important differences (MCIDs) and ≥ normative values (NVs) in HAQ-DI, SF-36 and FACIT-F (week 16 before EE) were analysed.

**Results:**

In total population, numerically higher improvements in most PROs were reported with abatacept (*n* = 213) versus placebo (*n* = 211) at both time points (*P* > 0.05). Higher proportions of abatacept versus placebo patients reported PRO improvements ≥ MCID and ≥ NV at week 16. At week 16, all PRO improvements were numerically greater (*P* > 0.05) in patients with baseline CRP > ULN versus CRP ≤ ULN (all significant [95% confidence interval] for abatacept vs placebo); improvements in SF-36 component summaries and FACIT-F were greater in TNFi-naïve versus TNFi-exposed patients (abatacept > placebo). Week 24 subgroup data were difficult to interpret due to low patient numbers.

**Conclusions:**

Abatacept treatment improved PROs in patients with PsA versus placebo, with better results in elevated baseline CRP and TNFi-naïve subpopulations.

**Trial registration:**

ClinicalTrials.gov number, NCT01860976 (funded by Bristol-Myers Squibb); date of registration: 23 May 2013.

**Electronic supplementary material:**

The online version of this article (10.1186/s13075-018-1769-7) contains supplementary material, which is available to authorized users.

## Background

Psoriatic arthritis (PsA) is a chronic inflammatory autoimmune disease with a range of clinical manifestations affecting skin and musculoskeletal systems [1]. Health-related quality of life (HRQoL) can vary greatly according to a patient’s specific symptoms; hence, assessing treatment effects using patient-reported outcomes (PROs) is particularly important in PsA [2–6]. Several PRO instruments have been validated in PsA, including the Health Assessment Questionnaire-Disability Index (HAQ-DI) [4, 7] and Short Form-36 (SF-36) [5, 6].

Abatacept, a selective T-cell co-stimulation modulator [8], has a distinct mechanism of action upstream of currently available agents, and is approved for treatment of rheumatoid arthritis and juvenile idiopathic arthritis, and recently for active PsA in adults [9]. In the phase 3 Active pSoriaTic aRthritis rAndomizEd triAl (ASTRAEA, NCT01860976), subcutaneous (SC) abatacept 125 mg weekly significantly increased the proportion of patients achieving ≥ 20% improvement in the American College of Rheumatology criteria (ACR20) compared with placebo at week 24 (primary endpoint: 39.4% vs 22.3%; *P* < 0.001) and was well tolerated in patients with active PsA [10]. A numerically higher proportion of patients with HAQ-DI responses (reductions from baseline ≥ 0.35) was evident with abatacept versus placebo (*P* > 0.05). Abatacept treatment also reduced progression of structural damage with an overall beneficial effect on musculoskeletal symptoms. However, due to the hierarchical testing procedure employed, it was not possible to attribute significance to endpoints ranked below HAQ-DI responses in the hierarchical testing [10].

The effect of factors associated with poor prognosis and treatment resistance, such as elevated C-reactive protein (CRP) levels and prior exposure to tumour necrosis factor inhibitors (TNFi) [11], was also evaluated in ASTRAEA. Higher ACR20 responses were observed with abatacept versus placebo in both TNFi-naïve and TNFi-exposed subpopulations at week 24, with the largest treatment differences seen in TNFi-naïve patients [10]. Moreover, patients with baseline CRP ≥ upper limits of normal (ULN) had the highest ACR20 responses at week 24 with abatacept versus placebo [10].

The goal of the analyses reported here was to examine the impact of abatacept versus placebo treatment on PROs in ASTRAEA for the overall population and in subgroups by baseline CRP levels and previous TNFi exposure.

## Methods

### Study design and treatment

The design, eligibility criteria, and main efficacy and safety endpoints of this phase 3, randomised, double-blind, placebo-controlled, multicentre trial have been reported in detail previously [10]. Patients were randomised (1:1) to receive SC abatacept 125 mg weekly or placebo for 24 weeks, after which all patients were transitioned to receive open-label SC abatacept weekly for 28 weeks (total study period of 52 weeks). Patients without ≥ 20% improvement in tender and swollen joint counts at week 16 were switched to open-label abatacept for 28 weeks (early escape [EE], total study period of 44 weeks). Key eligibility criteria included age ≥ 18 years, PsA per the Classification Criteria for PsA (CASPAR) [12], active arthritis (defined as ≥ 3 tender and ≥ 3 swollen joints), active plaque psoriasis with ≥ 1 qualifying target lesion ≥ 2 cm in diameter and inadequate response or intolerance to ≥ 1 non-biologic disease-modifying antirheumatic drug (DMARD). Both TNFi-naïve and TNFi-exposed patients were included.

### Patient-reported outcomes

HAQ-DI [4, 7], SF-36 physical component summary (PCS), mental component summary (MCS) and individual domain scores [5, 6], Functional Assessment of Chronic Illness Therapy-Fatigue scale (FACIT-F) [3] and Dermatology Life Quality Index (DLQI) [2] scores were assessed at weeks 16 and 24 in the overall population (prespecified) and in patient subpopulations (post hoc) by baseline CRP (> or ≤ ULN, defined as 3 mg/L) and prior TNFi use. The hierarchical order of the secondary and exploratory PRO endpoints [10] was predefined as: proportions of patients reporting HAQ-DI responses ≥ minimal clinically important differences (MCIDs) and mean changes from baseline in SF-36 PCS and MCS scores (summary and domain scores).

Here, in the overall population, the proportions of patients reporting improvements from baseline in HAQ-DI, SF-36 (summary and domain) and FACIT-F scores ≥ MCID (expressed as a value established for each instrument, and defined as the smallest change in score perceived by a patient to be clinically important) [13] and ≥ normative values (defined based on age/gender-matched population) were analysed (post hoc) at week 16 prior to confounding due to EE to open-label abatacept treatment. Defined MCIDs were: HAQ-DI ≥ − 0.35 [14], SF-36 PCS ≥ 2.5 [13, 15–17], SF-36 MCS ≥ 2.5 [13, 15, 17], SF-36 domains ≥5.0 [13, 15, 17], and FACIT-F ≥ − 4.0 [3]. Normative values were: HAQ-DI < 0.5 [7, 18, 19], SF-36 PCS ≥ 50 [17, 20], SF-36 MCS ≥ 50 [20] and FACIT-F ≥ 40.1 [21].

### Statistical analyses

All efficacy analyses included all randomised patients who received at least one dose of study medication (intent-to-treat population). Week 16, prior to EE, was the last time point at which all patients were analysed. For week 24 analyses, EE patient data were set to missing. As previously reported, the effect of abatacept on the first key secondary endpoint in the statistical hierarchy (HAQ-DI responses) did not reach significance; therefore, only nominal *P* values were generated for subsequent outcomes, which were ranked lower in the hierarchy. The significance of the treatment effect cannot be definitively attributed for these outcomes as they were not adjusted for multiplicity (however, 95% confidence intervals [CIs] were not overlapping) [10]. Nonetheless, these lower-ranking outcomes still provide a measure of clinical meaningfulness. Adjusted mean changes from baseline in PROs including SF-36 domain scores were evaluated, and corresponding adjusted mean differences (95% CI) between the abatacept and placebo groups were calculated using a longitudinal repeated measures model. This model included the fixed categorical effects of treatment, day, prior TNFi use, methotrexate (MTX) use, body surface area (BSA), day-by-treatment interaction, prior TNFi-use-by-day interaction, MTX-use-by-day interaction, BSA-use-by-day interaction and the continuous fixed covariate of baseline score and baseline score-by-day interaction. The estimate of difference (95% CI) between abatacept and placebo groups for MCID and normative values was calculated using a two-sided Cochran–Mantel–Haenszel chi-square test adjusted for stratification criteria.

### Patient consent and ethics approval

All patients or their legal representatives gave written, informed consent prior to study entry. The study was conducted in accordance with the Declaration of Helsinki, International Conference on Harmonisation Guidelines for Good Clinical Practice and local regulations. Schulman Associates Institutional Review Board or Independent Ethics Committees approved the protocol, consent form and any other written information provided to patients or their legal representatives.

## Results

### Patients

Of 424 patients randomised, 213 received abatacept and 211 placebo; 76 (35.7%) and 89 (42.2%), respectively, met criteria for EE [10]. Baseline demographic and disease characteristics were similar between treatment groups and were reported in detail previously [10].

### Overall population analysis

#### Changes from baseline at weeks 16 and 24

**Fig. 1:**
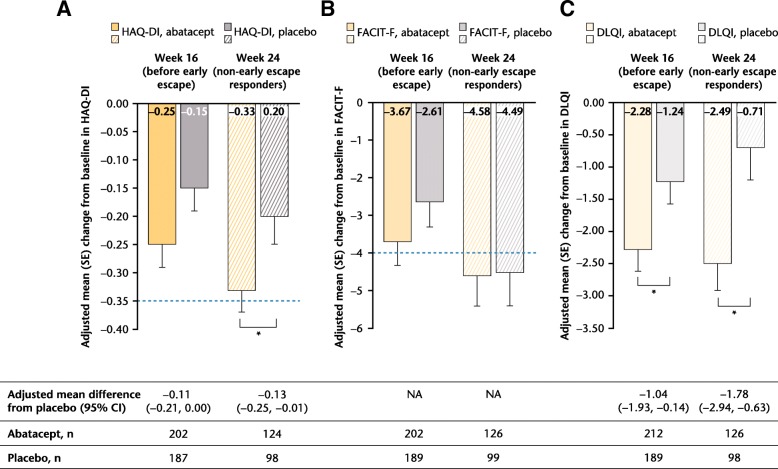
HAQ-DI (**a**), FACIT-F (**b**), DLQI (**c**) change from baseline (weeks 16 and 24, overall population). ^*^Statistically significant difference. *Dotted lines* represent MCID (HAQ-DI: ≥ − 0.35; FACIT-F: ≥ − 4.0). *CI* confidence interval, *DLQI* Dermatology Life Quality Index, *FACIT-F* Functional Assessment of Chronic Illness Therapy-Fatigue scale, *HAQ-DI* Health Assessment Questionnaire-Disability Index, *MCID* minimal clinically important difference, *NA* not applicable, *SE* standard error

**Fig. 2:**
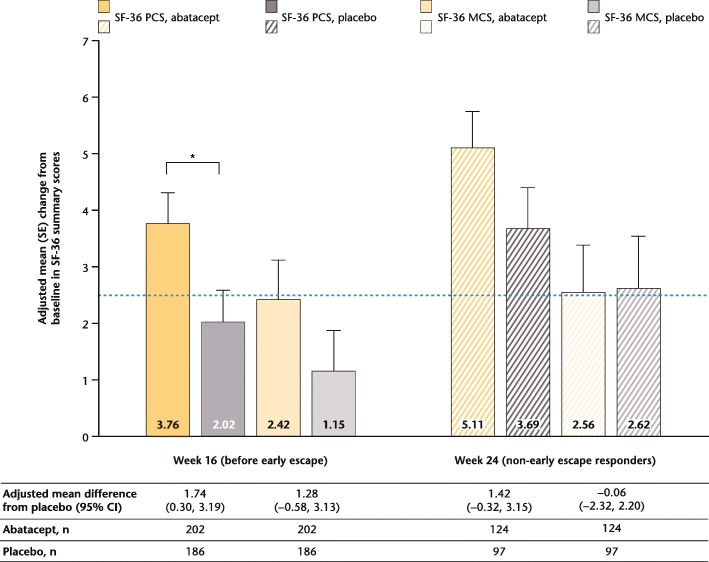
SF-36 PCS and MCS change from baseline (weeks 16 and 24, overall population). ^*^Statistically significant difference. *Dotted line* represents MCID (≥ 2.5). *CI* confidence interval, *MCID* minimal clinically important difference, *MCS* mental component summary, *PCS* physical component summary, *SE* standard error, *SF-36* Short Form-36

**Fig. 3:**
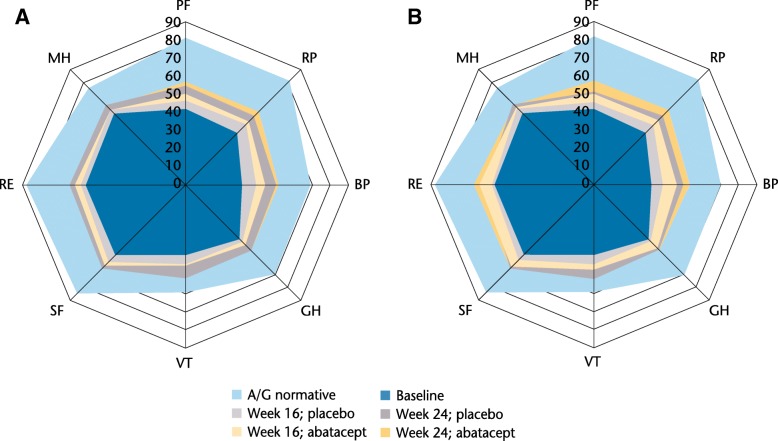
Abatacept/placebo SF-36 domain scores (baseline, weeks 16, 24) versus normative population (**a**, overall; **b**, CRP > ULN). Normative values for SF-36 individual domains were defined based on matching the age/gender distribution of this protocol population to US 1999 norms in patients without chronic disease or arthritis [20, 34]: PF and RP 81.9, BP 69.7, GH 70.4, VT 59.3, SF 84.4, RE 87.8, MH 75.6. *A/G* age/gender, *BP* bodily pain, *CRP* C-reactive protein, *GH* general health, *MH* mental health, *PF* physical function, *RE* role–emotional, *RP* role–physical, *SF* social function, *SF-36* Short-Form 36, *ULN* upper limit of normal, *VT* vitality

In the total population, greater improvements from baseline in most PROs were reported with abatacept versus placebo at both week 16, which comprised all patients, and week 24, which included only patients showing a response to either treatment (response defined as 20% improvement in tender and swollen joint counts; Figs. 1, 2 and 3a). Statistically significant (95% CI of difference vs placebo not crossing 0) improvements from baseline with abatacept versus placebo were reported in HAQ-DI scores in the week 24 responder group (Fig. 1a), in SF-36 PCS (Fig. 2), SF-36 physical functioning (PF), bodily pain (BP) and vitality (VT) domains (adjusted mean difference [95% CI], respectively: 4.44 [0.39 to 8.49], 5.36 [1.40 to 9.33] and 4.07 [0.67 to 7.47]), and in DLQI (Fig. 1c) scores at weeks 16 and 24.

Changes from baseline in SF-36 MCS scores were not statistically significant, but were numerically greater with abatacept versus placebo at week 16 (adjusted mean change from baseline [standard error (SE)]: 2.42 [0.70] vs 1.15 [0.73], adjusted mean difference [95% CI]: 1.28 [− 0.58 to 3.13]; *P* > 0.05), but were not meaningfully different for the responder-only analysis at week 24 (adjusted change from baseline [SE]: 2.56 [0.83] vs 2.62 [0.92], adjusted mean difference [95% CI]: –0.06 [− 2.32 to 2.20]). All SF-36 domains showed nonsignificant trends towards greater improvements from baseline with abatacept than placebo at week 16; improvements from baseline in all domains increased in both abatacept and placebo groups among responders at week 24 (Fig. 3a; see Additional file 1: Table S1).

#### Minimal clinically important differences and normative values

**Fig. 4:**
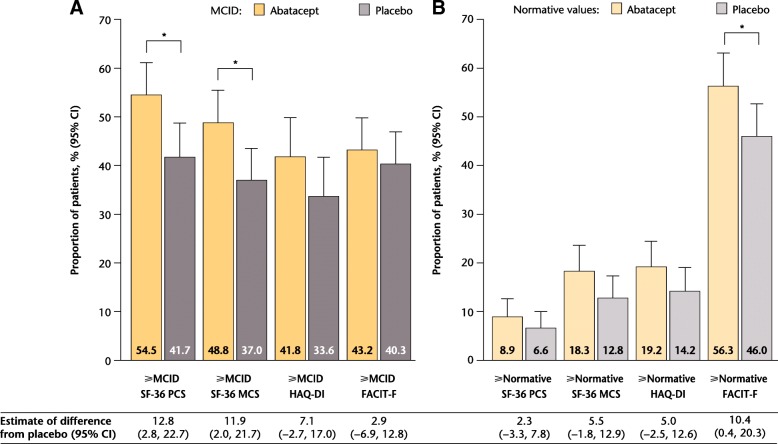
Rates of PRO improvements ≥ MCID (**a**) or ≥ normative values (**b**) at week 16 (overall population). ^*^Statistically significant difference. MCID values: HAQ-DI ≥ − 0.35, SF-36 PCS ≥ 2.5, SF-36 MCS ≥ 2.5, FACIT-F ≥ − 4.0 and SF-36 domains ≥5.0. Normative values: HAQ-DI ≥ 0.5, SF-36 PCS ≥ 50, SF-36 MCS ≥ 50 and FACIT-F ≥ 40.1. *CI* confidence interval, *FACIT-F* Functional Assessment of Chronic Illness Therapy-Fatigue scale, *HAQ-DI* Health Assessment Questionnaire-Disability Index, *MCID* minimal clinically important difference, *MCS* mental component summary, *PCS* physical component summary, *PRO* patient-reported outcome, *SF-36* Short Form-36

A statistically significant benefit at week 16 with abatacept versus placebo was evident in SF-36 PCS and MCS scores, and PF, BP and role–emotional (RE) domains (Fig. 4a; see Additional file 2: Table S2). The proportions of patients reporting scores ≥ normative values at week 16 were significantly greater (estimate of difference [95% CI]) with abatacept versus placebo in FACIT-F (10.4 [0.4 to 20.3]) and SF-36 RE domain (10.3 [3.4 to 17.1]) scores.

A numerically greater proportion of patients reported improvements ≥ MCID with abatacept versus placebo at week 16 in HAQ-DI scores, but the difference did not reach statistical significance (Fig. 4a). At week 16, the proportion of patients reporting improvements ≥ MCID in FACIT-F scores (Fig. 4a) and SF-36 role–physical (RP), general health (GH), VT, social function and mental health (MH) domain scores (see Additional file 2: Table S2) were numerically higher with abatacept versus placebo. In the abatacept treatment group, changes from baseline exceeded MCID in six of eight SF-36 domains, the exceptions being GH and MH; whereas in the placebo group, mean changes exceeded MCID in the RP and BP domains only.

The proportions of patients who reported scores ≥ normative values at week 16, although not statistically significant, were numerically higher with abatacept than placebo in HAQ-DI, SF-36 PCS and MCS, and FACIT-F (Fig. 4b) scores and all SF-36 domains (see Additional file 3: Table S3) (*P* > 0.05).

### Subpopulation analyses

#### Changes from baseline at weeks 16 and 24

**Fig. 5:**
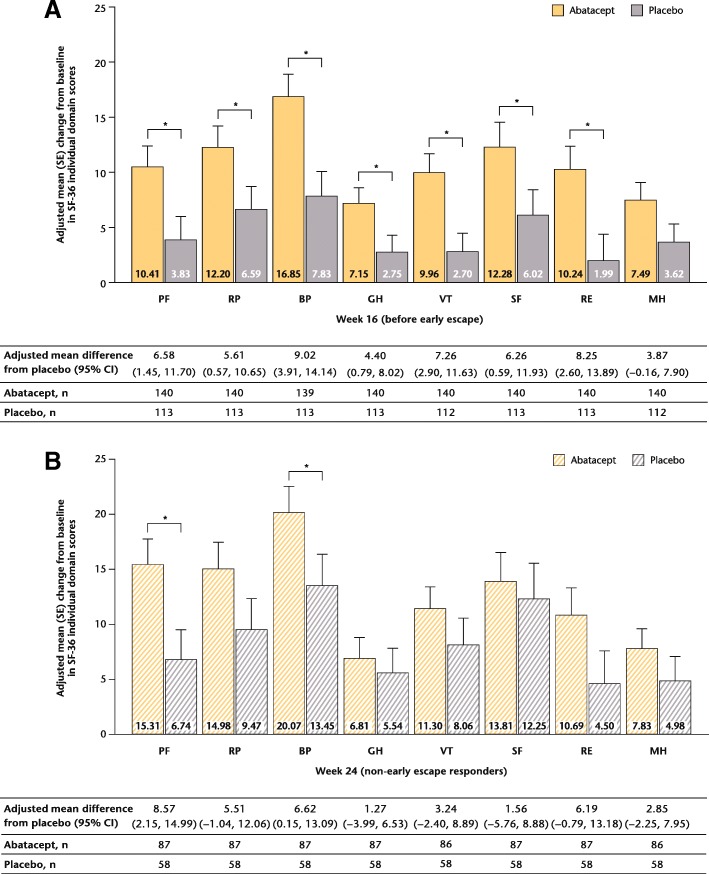
SF-36 domain score changes from baseline for CRP > ULN population: weeks 16 (**a**) and 24 (**b**). ^*^Statistically significant difference. *BP* bodily pain, *CI* confidence interval, *CRP* C-reactive protein, *GH* general health, *MH* mental health, *PF* physical function, RE role–emotional, *RP* role–physical, *SE* standard error, *SF* social function, *SF-36* Short Form-36, *VT* vitality

Across all PROs, improvements from baseline to week 16, although not statistically significant, were numerically greater in patients with baseline CRP > ULN versus those with CRP ≤ ULN in both abatacept and placebo groups (*P* > 0.05; Table 1). In the CRP > ULN subpopulation, improvements with abatacept versus placebo were significantly greater in HAQ-DI, SF-36 PCS, MCS, FACIT-F and DLQI scores (Table 1). Across all SF-36 domains, with the exception of MH, statistically significantly greater improvements were reported at week 16 with abatacept versus placebo in patients with baseline CRP > ULN (Fig. 3b and Fig. 5). Statistically significant improvements (adjusted mean difference [95% CI]) in DLQI (− 2.32 [− 3.80 to − 0.83]; Table 1) and SF-36 PF (8.57 [2.15 to 14.99]) and BP (6.62 [0.15 to 13.09]) (Fig. 5b) domain scores were reported in the baseline CRP > ULN subpopulation with abatacept versus placebo at week 24; however, data should be interpreted with caution due to low patient numbers. In the CRP ≤ ULN subpopulation, no significant improvements with abatacept versus placebo were evident at week 16 (Table 1).

**Table 1:** Adjusted mean change from baseline in PROs at weeks 16 (all patients) and 24 (non-EE responder analysis) in patients treated with abatacept or placebo and stratified by baseline CRP level or prior TNFi use

Subpopulation	Week 16 (before EE)			Week 24 (non-EE responders)		
CRP > ULN	Abatacept	Placebo	Adjusted mean difference (95% CI)	Abatacept	Placebo	Adjusted mean difference (95% CI)
HAQ-DI	−0.34 (0.05),^*^n = 139	−0.19 (0.05), *n* = 115	−0.15 (−0.28 to −0.02)	−0.41 (0.07), n = 86	−0.23 (0.08), *n* = 59	−0.18 (−0.37 to 0.01)
SF-36 PCS	4.97 (0.70),^*^n = 139	2.50 (0.75), *n* = 112	2.47 (0.63 to 4.31)	6.41 (0.83), *n* = 86	4.04 (0.95), *n* = 58	2.37 (0.13 to 4.61)
SF-36 MCS	4.54 (0.86),^*^n = 139	1.39 (0.94), *n* = 112	3.14 (0.85 to 5.44)	4.05 (1.05), *n* = 86	2.71 (1.24), *n* = 58	1.34 (−1.54 to 4.22)
FACIT-F	−5.19 (0.83),^*^n = 139	−2.81 (0.88), *n* = 116	−2.38 (−4.57 to −0.19)	−6.44 (1.03), *n* = 87	−5.44 (1.21), *n* = 60	−1.00 (−3.81 to 1.80)
DLQI	−2.72 (0.42),^*^n = 141	−1.48 (0.45), *n* = 116	−1.24 (−2.35 to −0.13)	−2.74 (0.55),^*^n = 87	−0.42 (0.64), *n* = 60	−2.32 (−3.80 to −0.83)
CRP ≤ ULN	Abatacept	Placebo	Adjusted mean difference (95% CI)	Abatacept	Placebo	Adjusted mean difference (95% CI)
HAQ-DI	−0.08 (0.06), *n* = 62	−0.08 (0.06), *n* = 70	0.01 (−0.14 to 0.15)	−0.12 (0.08), *n* = 37	−0.08 (0.08), *n* = 39	−0.04 (−0.26 to 0.19)
SF-36 PCS	1.33 (0.91), *n* = 62	1.56 (0.88), *n* = 72	−0.23 (−2.63 to 2.18)	3.04 (1.03), *n* = 37	3.10 (1.04), *n* = 39	−0.06 (−2.88 to 2.77)
SF-36 MCS	−1.95 (1.20), *n* = 62	0.85 (1.17), *n* = 72	−2.80 (−6.00 to 0.40)	−0.69 (1.29), *n* = 37	1.67 (1.31), *n* = 39	−2.37 (−5.94 to 1.21)
FACIT-F	−0.58 (1.03), *n* = 66	−2.47 (1.02), *n* = 78	1.89 (−0.86 to 4.63)	−0.77 (1.24), *n* = 38	−3.22 (1.28), *n* = 39	2.45 (−0.99 to 5.89)
DLQI	−1.28 (0.57), *n* = 63	−1.04 (0.56), *n* = 71	−0.24 (−1.77 to 1.28)	−1.60 (0.72), *n* = 38	−0.92 (0.75), *n* = 38	−0.69 (−2.70 to 1.33)
TNFi-naïve	Abatacept	Placebo	Adjusted mean difference (95% CI)	Abatacept	Placebo	Adjusted mean difference (95% CI)
HAQ-DI	−0.24 (0.06), *n* = 80	−0.15 (0.06), *n* = 75	−0.08 (−0.25 to 0.08)	−0.29 (0.06), *n* = 50	−0.17 (0.07), *n* = 35	−0.12 (−0.30 to 0.06)
SF-36 PCS	3.63 (0.89), *n* = 82	2.05 (0.91), *n* = 78	1.58 (−0.79 to 3.95)	4.70 (0.97), *n* = 49	2.92 (1.08), *n* = 34	1.78 (−0.91 to 4.46)
SF-36 MCS	2.98 (1.06),^*^ *n* = 82	−0.02 (1.08), *n* = 78	3.00 (0.19 to 5.81)	2.54 (1.17), *n* = 49	2.79 (1.38), *n* = 34	−0.25 (−3.60 to 3.10)
FACIT-F	−4.08 (1.01), *n* = 81	−2.17 (1.02), *n* = 79	−1.91 (−4.57 to 0.76)	−4.14 (1.21), *n* = 51	−4.65 (1.41), *n* = 35	0.51 (−0.29 to 3.93)
DLQI	−1.87 (0.51), *n* = 83	−0.92 (0.52), *n* = 79	−0.95 (−2.31 to 0.41)	−1.52 (0.67), *n* = 51	−0.23 (0.75), *n* = 34	−1.29 (−3.16 to 0.57)
TNFi-exposed^†^	Abatacept^‡^	Placebo^§^	Adjusted mean difference (95% CI)	Abatacept^‡^	Placebo^§^	Adjusted mean difference (95% CI)
HAQ-DI	−0.25 (0.05), *n* = 122	−0.13 (0.05), *n* = 112	−0.12 (−0.25 to 0.01)	−0.35 (0.06), *n* = 74	−0.18 (0.07), *n* = 63	−0.16 (−0.33 to 0.00)
SF-36 PCS	3.50 (0.70), *n* = 120	1.68 (0.74), *n* = 108	1.82 (−0.02 to 3.66)	5.02 (0.86), *n* = 75	3.70 (0.93), *n* = 63	1.32 (−0.95 to 3.59)
SF-36 MCS	2.03 (0.94), *n* = 120	1.97 (0.99), *n* = 108	0.06 (−2.41 to 2.53)	2.46 (1.16), *n* = 75	2.37 (1.24), *n* = 63	0.09 (−2.95 to 3.13)
FACIT-F	−3.34 (0.86), *n* = 121	−2.96 (0.89), *n* = 110	−0.39 (−2.64 to 1.86)	−4.61 (1.09), *n* = 75	−4.13 (1.16), *n* = 64	−0.48 (−3.34 to 2.38)
DLQI	−2.68 (0.45), *n* = 122	−1.61 (0.48), *n* = 110	−1.07 (−2.27 to 0.12)	−2.76 (0.59),^*^n = 75	−0.71 (0.63), *n* = 64	−2.04 (−3.59 to −0.49)

Data are adjusted mean change (SE) unless otherwise indicated. A positive change in SF-36 PCS and SF-36 MCS and a negative change in FACIT-F, HAQ-DI and DLQI corresponded to an improvement

*CI* confidence interval, *CRP* C-reactive protein, *DLQI* Dermatology Life Quality Index, *EE* early escape, *FACIT-F* Functional Assessment of Chronic Illness Therapy-Fatigue scale, *HAQ-DI* Health Assessment Questionnaire-Disability Index, *MCS* mental component summary, *PCS* physical component summary, *PRO* patient-reported outcome, *SE* standard error, *SF-36* Short Form-36, *TNFi* tumour necrosis factor inhibitor, *ULN* upper limit of normal

^*^95% CI of difference versus placebo did not cross 0

^†^At least 70% of the TNFi-exposed subpopulation were TNFi failures

^‡^Prior TNFi use, *n* (%): 1 TNFi, 94 (44.1); 2 TNFi, 31 (14.6); ≥ 3 TNFi, 4 (1.9)

^§^Prior TNFi use, *n* (%): 1 TNFi, 92 (43.6); 2 TNFi, 36 (17.1); ≥ 3 TNFi, 2 (0.9)

A statistically significant benefit for abatacept versus placebo was reported by TNFi-naïve patients at week 16 in SF-36 MCS (Table 1) scores. Among abatacept-treated TNFi-naïve patients at baseline, numerically, although not statistically significant, greater improvements in SF-36 PCS, MCS and FACIT-F scores at week 16 were reported versus TNFi-exposed patients (*P* > 0.05; Table 1). In the TNFi-naïve abatacept-treated subpopulation, adjusted mean changes from baseline at week 16 exceeded MCID in SF-36 PCS, MCS and FACIT- F scores (Table 1), and seven of eight SF-36 domains with exception of MH (data not shown). In TNFi-exposed abatacept-treated patients, improvements exceeded MCID in SF-36 PCS scores (Table 1).

## Discussion

These analyses demonstrated that abatacept treatment generally improved PROs in patients with active PsA in the phase 3 ASTRAEA trial, particularly in those who were TNFi-naïve and/or with elevated CRP at baseline*.* In the overall population at week 16, prior to EE, abatacept administration was associated with improved PROs compared with placebo; significant improvements with abatacept versus placebo were reported in SF-36 PCS, PF, BP and VT domain scores as well as DLQI, reflecting those areas of HRQoL most impacted by PsA. At week 24 in the non-EE responder analysis, a potential benefit of abatacept treatment was evident compared with placebo, with significantly greater improvements reported in physical function (by HAQ-DI) and dermatological manifestations (by DLQI). The proportion of patients with clinically meaningful HAQ-DI responses (reductions from baseline score ≥ 0.35) at week 24 was numerically higher with abatacept versus placebo: 31.0% versus 23.7%; however, as this did not reach statistical significance, it was not possible to definitively attribute significance to lower-ranking secondary endpoints in the hierarchical testing (nominal *P* values only were generated; 95% CIs were not overlapping) [10]. Notably, significant improvements in DLQI, the only PRO investigated here that directly measures the skin domain in PsA, were reported by those patients with a background of an overall modest skin response (by Psoriatic Area and Severity Index) in ASTRAEA at week 24 [10]. Nevertheless, as the week 24 analysis included only non-EE responders, the placebo arm comprised patients who reported responses to placebo. Therefore, it may be expected that differences between treatment groups would be less obvious at week 24 than week 16. In addition, the number of patients analysed at this time point was lower than at week 16.

Comparisons of the proportion of patients reporting improvements ≥ MCID is considered a clinically meaningful estimate of therapy effects [22]. Overall, the proportion of abatacept-treated patients reporting improvements ≥ MCID in PROs exceeded the proportion of placebo-treated patients: at week 16, 41.8–58.2% of abatacept-treated patients across different PROs reported clinically meaningful improvements in HAQ-DI, SF-36 PCS and MCS, individual SF-36 domains and FACIT-F scores compared with 33.6–47.9% of those treated with placebo.

In addition to the overall population analysis, PROs were analysed in subpopulations of patients by baseline CRP, as elevated CRP is an identified poor prognostic factor [11]. There was a non-statistically significant trend towards improved PROs in patients with elevated baseline CRP regardless of treatment arm at week 16. However, among patients with elevated CRP, those receiving abatacept reported greater improvements compared with placebo. Similarly, in the main ASTRAEA study, the highest ACR20 responses with abatacept versus placebo were seen in patients with CRP > ULN at baseline [10], suggesting that these patients may be particularly responsive to abatacept. Our results suggest that baseline CRP should be taken into consideration when evaluating the clinical efficacy of different treatments. PROs were also analysed in subpopulations by previous exposure to TNFi treatment. At week 16, improvements were greater with abatacept than with placebo in both TNFi-naïve and TNFi-exposed subpopulations. However, in abatacept-treated patients, reported improvements in PROs were generally larger in the TNFi-naïve versus TNFi-exposed subpopulations. Indeed, greater efficacy would be expected in TNFi-naïve than in TNFi-exposed patients [23]. These findings are in line with clinical outcomes observed with abatacept in this trial, which were generally better in patients with elevated CRP at baseline and TNFi-naïve patients [10]. The PRO data reported here support previous results that abatacept may be particularly effective in certain subpopulations of patients.

The effects of other DMARDs, including TNFi agents, on PROs in patients with active PsA have been investigated previously, with most studies assessing effects over 24 or 48 weeks. Statistically and clinically meaningful improvements in SF-36 PCS and MCS and all individual domain scores from baseline to week 24 have been reported with etanercept [24]; clinically meaningful improvements in PROs including HAQ-DI and SF-36 PCS scores have also been reported over 48 weeks of treatment [25]. Similarly, adalimumab has been shown to improve HRQoL, based on SF-36 PCS, HAQ-DI, FACIT-F and DLQI scores, after 48 weeks of treatment [26]. The effects of the newer interleukin inhibitors on PROs have also been studied. Ustekinumab, an anti-interleukin-12 and -23 agent, improved physical function (by SF-36 PCS and HAQ-DI) and dermatological manifestations (by DLQI) at week 24 [27, 28]. Beneficial effects on PROs have also been reported after 24 weeks with the anti-interleukin-17A agent secukinumab [29, 30]. Furthermore, apremilast, a phosphodiesterase 4 inhibitor, was shown to significantly improve HAQ-DI scores by week 16 compared with placebo in a 24-week trial in which < 10% of patients had previously failed a biologic therapy [31]. In the current study, improvements in PROs achieved with abatacept appeared less marked than that reported with other biologic DMARDs (bDMARDs) in earlier studies; however, differences in trial design and patient populations preclude comparisons of efficacy between abatacept and other bDMARDs based on currently available evidence.

The therapeutic options for PsA have greatly increased over the past 10 years and, as more new treatments are introduced, assessing responses to therapy including PROs will become increasingly important, aiding treatment choices. A recent literature review provided an evidence-based overview of 44 instruments per core PsA outcome domain to ascertain applicability and best instrument for each domain of the many available PROs [32]. However, further research is warranted to develop and validate specific PRO measures that better capture the impact of all PsA symptoms [33]. In the meantime, using a combination of instruments and/or the best available instrument per domain, as in this trial, provides a more complete picture [33].

A number of study limitations should be considered. First, subpopulation comparisons and ascertainment of scores ≥ MCID and ≥ normative values were post hoc in nature. Second, owing to the particular trial design, a high proportion of patients were subject to EE at week 16; as such, week 24 analyses included a limited number of patients who were still receiving blinded treatment in either arm of the trial. Only nominal *P* values were provided for endpoints that ranked lower in the statistical hierarchy than the first secondary endpoint, which did not reach statistical significance at the 5% level. For other endpoints, only 95% CIs of differences between abatacept and placebo arms were generated, without associated *P* values. In addition, due to the low patient numbers, the reported data for subpopulations were difficult to interpret, particularly at week 24. Finally, certain PROs may improve less rapidly over time and thus the week 16 time point may have not allowed maximal effects of abatacept treatment to be observed. In addition, although some statistically significant improvements were noted with abatacept, these may not necessarily be clinically important.

## Conclusions

In conclusion, abatacept treatment improved PROs at week 16 in patients with active PsA, with evidence of some effects sustained at week 24. Furthermore, PRO improvements were greater in TNFi-naïve patients and those with elevated CRP. These results demonstrate that clinical improvements in PsA signs and symptoms previously reported with abatacept treatment [10] also result in clinically meaningful improvements in PROs.

## Additional files

Additional file 1: **Table S1.** Adjusted mean change from baseline in SF-36 individual domains at weeks 16 (all patients) and 24 (non-EE responder analysis) in patients treated with abatacept or placebo (overall population). (DOCX 36 kb)

Additional file 2: **Table S2.** Proportion of patients (95% CI) treated with abatacept or placebo reporting improvements ≥MCID in SF-36 individual domains at week 16 (all patients) in the overall population. (DOCX 14 kb)

Additional file 3: **Table S3.** Proportion of patients (95% CI) treated with abatacept or placebo reporting improvements ≥normative values in SF-36 individual domains at week 16 (all patients) in the overall population. (DOCX 14 kb)

## Abbreviations

ACR20: American College of Rheumatology criteria
ASTRAEA: Active pSoriaTic aRthritis rAndomizEd triAl
bDMARDS: Biologic disease-modifying anti-rheumatic drugs
BP: Bodily pain
BSA: Body surface area
CASPAR: Classification Criteria for Psoriatic Arthritis
CI: Confidence interval
CRP: C-reactive protein
DLQI: Dermatology Life Quality Index
DMARD: Disease-modifying antirheumatic drug
EE: Early escape
FACIT-F: Functional Assessment of Chronic Illness Therapy-Fatigue
GH: General health
HAQ-DI: Health Assessment Questionnaire-Disability Index
HRQoL: Health-related quality of life
MCID: Minimal clinically important difference
MCS: Mental component summary
MH: Mental health
MTX: Methotrexate
NV: Normative values
PCS: Physical component summary
PF: Physical functioning
PRO: Patient-reported outcome
PsA: Psoriatic arthritis
RE: Role–emotional
RP: Role–physical
SC: Subcutaneous
SE: Standard error
SF-36: Short Form-36
TNFi: Tumour necrosis factor inhibitor
ULN: Upper limit of normal
VT: Vitality
