# Supplementary material for: Improved patient-reported outcomes in patients with psoriatic arthritis treated with abatacept: results from a phase 3 trial
Source: Arthritis Res Ther. 2018 Dec 6;20:269. doi: 10.1186/s13075-018-1769-7 (PMC6282264; doi:10.1186/s13075-018-1769-7)
Supplement: Supplementary file 1 — Table S1. Adjusted mean change from baseline in SF-36 individual domains at weeks 16 (all patients) and 24 (non-EE responder analysis) in patients treated with abatacept or placebo (overall population). (DOCX 36 kb) [file 13075_2018_1769_MOESM1_ESM.docx]

**Table S1** Adjusted mean change from baseline in SF-36 individual domains at weeks 16 (all patients) and 24 (non-early escape responder analysis) in patients treated with abatacept or placebo (overall population)

| **SF-36 domain** | **Week 16 (before early escape)** | | | **Week 24 (non-early escape responders)** | | |
| --- | --- | --- | --- | --- | --- | --- |
|  | **Abatacept** | **Placebo** | **Adjusted mean difference (95% CI)** | **Abatacept** | **Placebo** | **Adjusted mean difference (95% CI)** |
| **Physical function** | 7.90 (1.53),*  n=203 | 3.47 (1.59),  n=187 | 4.44  (0.39 to 8.49) | 12.08 (1.77),  n=125 | 7.31 (1.99),  n=97 | 4.77  (−0.09 to 9.63) |
| **Role–physical** | 8.84 (1.54),  n=203 | 5.42 (1.59),  n=187 | 3.42  (−0.64 to 7.48) | 11.56 (1.88),  n=125 | 7.96 (2.10),  n=97 | 3.60  (−1.54 to 8.74) |
| **Bodily pain** | 12.21 (1.51),*  n=202 | 6.85 (1.55),  n=187 | 5.36  (1.40 to 9.33) | 15.63 (1.84),  n=125 | 12.52 (2.06),  n=97 | 3.11  (−1.92 to 8.13) |
| **General health** | 4.35 (1.12),  n=203 | 2.12 (1.16),  n=187 | 2.23  (−0.72 to 5.19) | 4.46 (1.46),  n=125 | 5.56 (1.62),  n=97 | −1.10  (−5.09 to 2.89) |
| **Vitality** | 6.16 (1.28),*  n=203 | 2.09 (1.33),  n=186 | 4.07  (0.67 to 7.47) | 8.23 (1.57),  n=124 | 7.53 (1.76),  n=97 | 0.70  (−3.61 to 5.01) |
| **Social function** | 7.38 (1.66),  n=203 | 4.14 (1.73),  n=187 | 3.24  (−1.15 to 7.64) | 9.39 (2.00),  n=125 | 9.39 (2.24),  n=97 | 0.00  (−5.48 to 5.48) |
| **Role–emotional** | 6.04 (1.70),  n=203 | 2.17 (1.77),  n=187 | 3.87 (−0.62 to 8.36) | 7.56 (2.00),  n=125 | 4.99 (2.25),  n=97 | 2.57 (−2.91 to 8.05) |
| **Mental health** | 4.62 (1.20),  n=203 | 3.13 (1.26),  n=186 | 1.48 (−1.70 to 4.67) | 5.03 (1.43),  n=124 | 4.91 (1.61),  n=97 | 0.12 (−3.79 to 4.03) |

Data are adjusted mean change (SE) unless otherwise indicated.

*95% CI of difference versus placebo did not cross 0.

CI, confidence interval; SE, standard error; SF-36, Short Form-36.
