# Supplementary material for: Improved patient-reported outcomes in patients with psoriatic arthritis treated with abatacept: results from a phase 3 trial
Source: Arthritis Res Ther. 2018 Dec 6;20:269. doi: 10.1186/s13075-018-1769-7 (PMC6282264; doi:10.1186/s13075-018-1769-7)
Supplement: Supplementary file 2 — Table S2. Proportion of patients (95% CI) treated with abatacept or placebo reporting improvements ≥MCID in SF-36 individual domains at week 16 (all patients) in the overall population. (DOCX 14 kb) [file 13075_2018_1769_MOESM2_ESM.docx]

**Table S2** Proportion of patients (95% CI) treated with abatacept or placebo reporting improvements ≥MCID in SF-36 individual domains at week 16 (all patients) in the overall population

| **SF-36 domain** | **Abatacept (n=213)** | **Placebo (n=211)** | **Estimate of difference (95% CI)** |
| --- | --- | --- | --- |
| **Physical function** | 58.2 (51.6 to 64.8)* | 47.4 (40.7 to 54.1) | 10.8 (0.9 to 20.7) |
| **Role–physical** | 53.5 (46.8 to 60.2) | 47.9 (41.1 to 54.6) | 5.7 (−4.3 to 15.6) |
| **Bodily pain** | 56.3 (49.7 to 63.0)* | 45.0 (38.3 to 51.7) | 11.3 (1.4 to 21.2) |
| **General health** | 48.4 (41.6 to 55.1) | 41.2 (34.6 to 47.9) | 7.1 (−2.8 to 17.0) |
| **Vitality** | 51.2 (44.5 to 57.9) | 42.7 (36.0 to 49.3) | 8.5 (−1.4 to 18.5) |
| **Social function** | 46.9 (40.2 to 53.7) | 38.9 (32.3 to 45.4) | 8.1 (−1.8 to 17.9) |
| **Role–emotional** | 49.3 (42.6 to 56.0)* | 36.0 (29.5 to 42.5) | 13.3 (3.5 to 23.1) |
| **Mental health** | 51.6 (44.9 to 58.4) | 43.1 (36.4 to 49.8) | 8.5 (−1.4 to 18.5) |

Data are % (95% CI).

*95% CI of difference versus placebo did not cross 0.

CI, confidence interval; MCID, minimal clinically important difference; SF-36, Short Form-36.
