# Supplementary material for: Improved patient-reported outcomes in patients with psoriatic arthritis treated with abatacept: results from a phase 3 trial
Source: Arthritis Res Ther. 2018 Dec 6;20:269. doi: 10.1186/s13075-018-1769-7 (PMC6282264; doi:10.1186/s13075-018-1769-7)
Supplement: Supplementary file 3 — Table S3. Proportion of patients (95% CI) treated with abatacept or placebo reporting improvements ≥normative values in SF-36 individual domains at week 16 (all patients) in the overall population. (DOCX 14 kb) [file 13075_2018_1769_MOESM3_ESM.docx]

**Table S3** Proportion of patients (95% CI) treated with abatacept or placebo reporting improvements ≥normative values in SF-36 individual domains at week 16 (all patients) in the overall population

| **SF-36 domain** | **Abatacept (n=213)** | **Placebo (n=211)** | **Estimate of difference (95% CI)** |
| --- | --- | --- | --- |
| **Physical function** | 13.1 (8.6 to 17.7) | 9.5 (5.5 to 13.4) | 3.7 (−2.8 to 10.2) |
| **Role–physical** | 8.9 (5.1 to 12.7) | 5.2 (2.2 to 8.2) | 3.7 (−1.6 to 9.0) |
| **Bodily pain** | 14.1 (9.4 to 18.8) | 8.5 (4.8 to 12.3) | 5.6 (−0.9 to 12.0) |
| **General health** | 9.9 (5.9 to 13.9) | 7.1 (3.6 to 10.6) | 2.8 (−3.0 to 8.5) |
| **Vitality** | 17.4 (12.3 to 22.5) | 15.2 (10.3 to 20.0) | 2.2 (−5.3 to 9.7) |
| **Social function** | 18.8 (13.5 to 24.0) | 12.3 (7.9 to 16.8) | 6.5 (−0.9 to 13.8) |
| **Role–emotional** | 18.3 (13.1 to 23.5)* | 8.1 (4.4 to 11.7) | 10.3 (3.4 to 17.1) |
| **Mental health** | 15.0 (10.2 to 19.8) | 10.0 (5.9 to 14.0) | 5.1 (−1.7 to 11.8) |

Data are % (95% CI).

*95% CI of difference versus placebo did not cross 0.

CI, confidence interval; SF-36, Short Form-36.
